# Obstetrics and Gynecology

**Published:** 1893-10

**Authors:** 


### OBSTETRICS AND GYNECOLOGY.

---

EDITED BY DR. VIRGIL O. HARDON.

---

### THE SO-CALLED CONSERVATIVE OPERATIONS UPON THE OVARIES, TUBES AND UTERUS.

The author emphasizes the points that removal of both adnexa is a procedure frequently of questionable utility, that most of the doubts expressed with reference to the conservative method are unwarranted, and that the results of the operation in a large majority of cases are exceptionally favorable.

1. *Resection of the Ovary.*—It is a well known fact that while both ovaries are usually found simultaneously affected, the disease in both is not always due to the same cause; the one ovary may be in a condition of destruction from a new growth, while the other may be the seat of cystic degeneration; in the one there may be suppurating follicles and abscesses, and in the other circumscribed cysts. Dropsy of the follicles was cured long ago by Spencer Wells by simple puncture, and excision of a degenerated segment of ovary was first performed in a systematic manner by Schroeder. Notwithstanding these favorable results, the majority of gynecologists are in favor of complete removal of partially diseased ovaries. Dr. Martin thinks this necessary in cases where the portion of ovary unaffected by the disease is incapable of function, and where a suppurative process exists, no matter what be the cause of the pus formation. In the minority of cases, however, where there is present chronic oöphoritis or circumscribed neoplasms, he regards this as unnecessary. Among twenty-seven cases in which he has operated by conservative methods, one died of infection from the suppurating tumor on the other side. Of the remaining twenty-six cases, recent reports are at hand from twenty-four, and only two of these have suffered recurrences. The fear that the remaining portion of the ovary will again become diseased is therefore unfounded. But even if a greater percentage than two out of twenty-four cases had become recurrent, this would be of little significance in view of the fact that eight of the patients subsequently gave birth to children. In five of these eight cases resection was performed for chronic oöphoritis and in three for small cystic growths. Five of the patients became pregnant for the first time after the operation. P. Muller advocates ignipuncture for the destruction of small cysts as a substitute for resection, and Pozzi also expresses himself very favorably regarding ignipuncture. His results in six cases of resection and six of ignipuncture were excellent, but they are of too recent date to warrant conclusions with reference to recurrences and pregnancy. Martin has been so successful with puncture, or excision and suture, that he considers ignipuncture an unnecessary complication.

2. *Resection of Occluded Tubes.*—It is much more difficult in the case of diseased tubes to determine the character of the disease. Great caution must be exercised, and in many cases where the contents of the obstructed tube seemed somewhat suspicious, Martin has resorted, in place of the anticipated resection, to complete removal of the tube and of the frequently healthy ovary. If the contents are turbid or purulent, if the mucous membrane is ulcerated, he abstains from resection. If, however, the mucous membrane is smooth—a condition frequently not determined until after washing away the adherent secretions—and if fluid contents are not found, then resection is justifiable. Among forty cases treated by the author, thirty-eight were cured by resection. As these cases presented severe complications, a mortality of five per cent. cannot be considered unfavorable. The objections urged against the opening of occluded tubes and the establishment of a new infundibulum have not been realized by Martin's experience, at least those according to which this operation favors a fresh infection or development of an extrauterine pregnancy. Of the thirty-eight patients who recovered, only four were not permanently cured or improved, and in view of the fact that all the cases were complicated by peritonitis, this percentage of cures is comparatively high. Only one of the patients became pregnant, but twelve were unmarried, and among the married ones nearly one-half of the husbands were neurasthenic, or suffered from chronic gonorrhea. Martin thinks that even if, in his cases of tubal resection, pregnancy should not occur, the removal of the disturbances and the improvement of the general health in the majority of patients must be regarded as a decided success.—*By Prof. A. Martin, Berlin.*

---

### HYDRASTININE IN UTERINE HEMORRHAGE.

Gottschalk, *Brooklyn Medical Journal*, says hydrastinine may be employed:

1. First of all, in those uterine hemorrhages which are traceable to a pronounced congestion of the uterus. To these belong, above all, the often very profuse menorrhagias of spinsters, in whom there is no pathological change in the condition of the genitals. In some of these cases it is possible to obtain a permanent result, so that even after discontinuing the remedy the menstrual flow remains smaller.

2. Also in hemorrhages which have their pathological and anatomical cause in endometritis, hydrastinine will lessen the quantity of blood; but here, according to Gottschalk's experience, the action is only palliative, not being sufficient alone to cure the local cause of the trouble.

3. For prophylactic or intermenstrual use, hydrastinine is useful before or during the first returning profuse menstruation after an abrasion of the uterine mucosa. It is well known that this menstruation, usually occurring after six weeks, is often very profuse. In the very cases where there was a great loss of blood before the operation, it is of great importance to prevent further profuse hemorrhage. This is possible if the treatment with hydrastinine is begun several days before the expected menstruation, and, if necessary, continued during the duration of the menstruation.

4. Menorrhagias caused by retroflexio uteri are best treated by correction of the malposition; but for cases of fixed retroflexion, where the reposition is not yet possible, hydrastinine is a commendable remedy.

5. Secondly uterine hemorrhages—*i. e.*, those caused by a change of the adnexa and their surroundings—offer a large field for the successful use of hydrastinine. To these belong the menorrhagia and metrorrhagia with pyosalpinx, oöphoritis, ovarian tumors and exudations. Of course the cause of the trouble is not influenced by the remedy.

6. Climacteric menorrhagias are much diminished by a faithfully carried out hydrastinine treatment.

---

### PALLIATIVE TREATMENT OF UTERINE CANCER.

Dr. Lucas Championniere recommends the following treatment: In cases of purulent discharges injections of solutions of permanganate of potash or salol, as hot as can be borne, should be employed. Stronger, irritant or poisonous antiseptics should be avoided. Immediateiy after injection the cancer is to be sprinkled with a mixture in equal parts of benzoin, iodoform, and magnesium carbonate. In cases of hemorrhage hot vaginal injections are likewise indicated, followed by tamponing with aseptic gauze. To prevent hemorrhages, curetting followed by application of the thermocautery is of value. After this operation, the parts should be covered daily with the above mentioned antiseptic and drying powder until the eschar has been detached.—*Rev. Gen. de Clinique et de Therap.*, No. 21, 1893.

---

### SURGICAL TREATMENT OF CRANIAL INFRACTION IN A NEW-BORN INFANT.

Dr. I. W. Smith, Charles City, Iowa, says: Cranial injuries are liable to occur independently of undue or badly applied instrumental force. The writer has used forceps rather freely and frequently for a country practitioner, and until a few months ago never injured a child. Then came an instance where a fronto-parietal depression was attributed to a bad use of the instrument. The infant developed obscure symptoms and perished after about twelve days. Not long after this experience I delivered Mrs. J. S. with forceps for the third time. The woman is a dwarf, but her two previous deliveries were accomplished without any untoward event. The presentation was right occipito-anterior. The application was high, and the impingements of the tips of the blades of the Hodge instrument were on the left frontal eminence and over the right ear. During extraction a sense of something giving way was felt for a moment, and on emergence of the head there was found a depression of the cranium on the right side of frontal bone. The main axis of depression was a line starting from a point on the sagittal suture a little back of its junction with the coronal, and running forward, downward, and outward a distance of about three inches. The breadth of the depression was about two inches, and its greatest depth not less than half an inch.

The child was large and vigorous, but it hardly slept or nursed and was restless and crying constantly. On the fourth day it was decided to treat the case on general principles. The infant was anesthetized, the scalp opened, and the bone divided by Hey's saw transversely to the depression axis. The bone was lifted by the elevator applied successively on either side of the saw track. A slender, long spicule was removed. There was some free venous hemorrhage, but no shock, and the child slept and nursed normally from the hour of the operation. Primary union followed and a month later the infant seems perfectly well. I believe the injury was caused by unskillful application of force, bringing the injured part across the promontory of a strongly curved sacrum.—*Exchange.*

---

### THE PATHOLOGY AND TREATMENT OF INJURIES OF THE PELVIC FLOOR.

Skene (*American Gynecological Journal*, May, 1893) divides all the injuries that are sustained in the pelvic floor by labor into two classes: (1) Those that occur in the median line of the floor and in a direction corresponding with the axis of the pelvis, and (2) injuries which occur above the floor itself—transverse, internal lacerations.

The former occur in various forms and degrees: (*a*) a solution of continuity of all the tissues, extending from the posterior commissure to the sphincter ani; (*b*) the same injury as the above, plus laceration of the sphincter. To these Skene would add subcutaneous lacerations of the muscles and fascia in the median line usually limited to the transversus perinei muscle and fascia, but in rare cases involving the anal sphincter. The so-called rectocele which is said to follow these injuries the author believes not to be a rectocele at all, but a prolapse of the vaginal wall and a varicose condition of the veins lying between the vagina and rectum, just within or above the pelvic floor.

In the treatment of these conditions the author suggests for injuries in the median line, extending from the posterior commissure to and including all the tissues of the pelvic floor at this point, the simple removal of the scar tissue, thus vivifying the ends of the muscles and fascia that have been divided. The vaginal wall is freed and forms the inner surface of the pelvic floor, while the lateral surfaces are united with sutures. In complete lacerations involving the sphincter ani, he follows closely Emmet's operation. For subcutaneous lacerations in the median line a form of flap-splitting operation is indicated.

### The Binder in Childbed.

Windmüller (*Centralbl. f. Gynäk.*, No. 27, 1893) recently opened a long and instructive discussion on the management of normal labor and childbed. He had conducted over a thousand labors in private practice alone in the course of thirty years. He wraps flannel round the body, but objects to the abdominal binder, which he finds interferes with the functions of the intestines, checks involution of the uterus, and is in the way during ablution. For the first three days he limits diet to milk, bread and butter, rice, spinach, potatoes and fish. He forbids wine and coffee, and believes, with Zweifel, that errors in diet are amongst the chief causes of prominent abdomen. The patient is kept on her back for three days, but Windmüller holds that ten days rest in bed is sufficient. He has had no cases of prominent abdomen in any of his patients, even in multiparæ, with the exception of those who had grown fat. In the discussion several experienced obstetricians differed as to whether prominent abdomen was due to deposit of fat in the parietes after labor, or to distention of the intestines which could be controlled by the binder. Many surgeons applied no bandage after abdominal section; but the physiological conditions were then not the same as in the puerperium. Schrader and others believed that the binder did more good than harm; he laid great stress on diet in childbed as influencing the future form of the abdomen. All food liable to cause flatulence must be avoided; spinach and potatoes were particularly bad in this respect. When the pelvis was narrow, or its angles too acute, the chances of the abdomen becoming pendulous after labor were great. To counteract the evil a good abdominal binder should be worn during the last months of pregnancy. In Japan it was customary for women to wear a binder during the entire second half of gestation. The binder in pregnancy supported the anterior walls, and greatly diminished the chances of parting of the recti.—*British Medical Journal.*—*Lancet-Clinic.*

